# A Retrospective Media Content Analysis of Suicide Reporting in Nigerian Print Media

**DOI:** 10.7759/cureus.92406

**Published:** 2025-09-15

**Authors:** Udoka Okpalauwaekwe, Kenneth E Enwerem, Moses Audu, Mansfield Mela

**Affiliations:** 1 Academic Family Medicine, University of Saskatchewan, Saskatoon, CAN; 2 Surgery, Faith Alive Foundation, Jos, NGA; 3 Public Health, Omega-Cares Foundation, Jos, NGA; 4 Psychiatry, Jos University Teaching Hospital, Jos, NGA; 5 Psychiatry, University of Saskatchewan, Saskatoon, CAN

**Keywords:** gender disparities, media reporting, nigeria, suicide behaviours, suicide prevention, suicide reporting, who guidelines

## Abstract

Suicide is a significant global public health concern. Nigeria, Africa’s most populous country, faces distinct challenges in suicide reporting due to underreporting, deep-rooted stigma, and the criminalization of attempted suicide. This study examines how Nigerian print media portrays suicide and evaluates adherence to World Health Organization (WHO) guidelines for responsible suicide reporting. We conducted a systematic review using Preferred Reporting Items for Systematic reviews and Meta-Analyses (PRISMA) guidelines. Suicide-related reports published between January 2010 and December 2021 across seven national Nigerian newspapers were analyzed. Data were coded for suicide methods, demographic characteristics, geographic distribution, and compliance with WHO suicide reporting standards. Descriptive analyses were conducted using IBM SPSS Statistics for Windows, version 28 (IBM Corp., Armonk, New York, United States). A total of 342 suicide events involving 425 individuals were identified; of these, 281 (66.3%) were male and 144 (33.6%) female. Suicide bombing occurred in 145 (42.3%) events, followed by hanging in 87 (25.4%) and poisoning in 55 (16.0%). Suicide events were more concentrated in the North-East zone (n=109, 33.0%) and least in the North Central zone. Media adherence to WHO reporting guidelines was extremely poor: 341/342 (99.7%) reports omitted preventive education/helplines, 341/342 (99.7%) showed sensationalist framing, 259/342 (75.7%) repeated “suicide” prominently, 339/342 (99.1%) detailed method/location; 325/342 (95.0%) showed limited consideration for the bereaved, and 91/342 (26.6%) included photographs. This study reveals concerning gaps in how Nigerian print media report suicide, with widespread neglect of WHO guidelines. Improved media practices are essential for ethical journalism and effective suicide prevention. Responsible reporting can enhance public understanding and reduce stigma, contributing to national mental health improvement. Our study findings underscore an urgent call for Nigeria to transform its media landscape into a strategic ally in suicide prevention, where accurate, sensitive reporting saves lives rather than sensationalizes tragedy.

## Introduction and background

Suicide is a significant global public health concern, affecting individuals across all age groups and socio-economic backgrounds [1]. It is defined as the deliberate and intentional act of ending one’s own life [2,3]. According to the World Health Organization (WHO), an estimated 703,000 people die by suicide globally each year, and for every suicide, there are many more people who attempt it or experience suicidal ideation [4]. Suicide remains among the top four leading causes of death among individuals aged 15-29 years, highlighting the urgent need for global preventive strategies [4].

In Africa, the second-most populous continent, Nigeria stands out due to its large and rapidly growing population, currently estimated at over 223 million people [5]. Despite this demographic significance, suicide research in Nigeria is hampered by major structural and cultural barriers [2,6]. Official suicide statistics submitted to WHO are often incomplete, derived from limited hospital records or specific case studies, and likely underestimate the true burden of suicide [2,7]. Cultural taboos, religious interpretations, and social stigma contribute to the misclassification, concealment, or outright denial of suicide cases [8,9]. Furthermore, Section 327 of Nigeria’s Criminal Code criminalizes suicide attempts, punishable by up to one year in prison, which not only hinders accurate reporting but also discourages individuals from seeking help [10].

Previous WHO estimates placed Nigeria’s suicide rate at 17.3 per 100,000 in 2016, one of the highest in sub-Saharan Africa [1,11,12]. While more recent estimates suggest rates ranging from 12.5 to 14.3 per 100,000, these figures remain subject to significant underreporting [6]. Nigeria has been ranked among the top 15 countries globally for suicide burden and continues to face unique challenges due to complex social, political, and religious dynamics [1,4,13].

The underlying causes of suicide in Nigeria are multifactorial [14-19]. Major depression, substance use disorders, psychological trauma, and developmental challenges are frequently cited, often exacerbated by economic instability, unemployment, domestic violence, stigma, and limited access to mental health services [8,20]. These challenges are compounded by a fragile mental health infrastructure. In addition, cultural and religious interpretations of suicide often fuel stigma and silence, reducing the likelihood of help-seeking behavior. Studies have shown that while women may experience higher rates of suicidal ideation, men are more likely to complete suicide, often using more violent methods such as hanging, firearms, or ingestion of toxic substances, resulting in a male-to-female suicide ratio of approximately 3:1 [1,7,20]. These gendered patterns of suicide risk reflect not only method lethality but also entrenched social norms that discourage emotional vulnerability in men, while penalizing help-seeking in both genders.

The media also plays a powerful role in shaping public perception and social norms surrounding mental illness and suicide [21-23]. Media portrayals can either contribute to public awareness and prevention or worsen stigmatization and suicide contagion, particularly through irresponsible or sensationalist reporting. In Nigeria, anecdotal and emerging evidence suggests that certain types of media coverage (especially in print and online platforms) have reinforced stereotypes, linked suicide to shame or spiritual retribution, and amplified fear rather than education in rural and semi-urban areas [19]. Recognizing this, the WHO developed specific media guidelines to promote responsible reporting on suicide. These guidelines discourage sensationalism, promote the use of helpline information, avoid detailed descriptions of suicide methods, and encourage compassion toward the bereaved and at-risk individuals [1].

Study Objectives 

This review study critically examines how Nigerian print media portray suicide events and adhere to the WHO guidelines for suicide reporting when covering such incidents.

## Review

Methodology and methods

Design

We applied a systematic approach to searching and critically reviewing literature using the Preferred Reporting Items for Systematic reviews and Meta-Analyses (PRISMA) as a reporting guideline [24] to ensure rigour and reproducibility of findings.

Protocol Registration and Reporting Information

There was no pre-published or registered protocol before the commencement of this study. 

Data Sources

We focused our data sources solely on Nigerian media portals (newspapers), chosen based on their national coverage estimates and credibility. The decision to focus on print media only was made to ensure consistency and reproducibility in data collection, as print sources offer stable archives and structured formats suitable for systematic analysis. We selected seven prominent media outlets as data sources to investigate the research question: how Nigerian print media’s reporting on suicide aligns and compares with the WHO guidelines for suicide prevention. These media houses included the following: *Daily Trust*, *Guardian*, *The Nation*, *Punch*, *This Day*, *Tribune*, and *Vanguard*.

Eligibility Criteria

We identified a priori to include newspapers that reported in English as well as in local languages (e.g., Pidgin English), and were published between January 1, 2010, and December 31, 2021. We did not include reports of attempted suicides unless there was clear suicidal intent and they were part of a completed event or multiple-victim scenario.

Information Sources and Search Strategy

With the assistance of a reference librarian at the National Library, Abuja, Nigeria, we identified keywords and synonyms for two major areas: “suicide” and “Nigeria”. Synonyms included “deliberate self-harm”, “self-injurious behavior”, “risk of suicide”, “self-killing”, “parasuicide”, “suicide bombing”, and “attempted suicide”. Newspapers were keyword-searched and selected for screening and inclusion using the search strategy and eligibility criteria. 

Selection of Sources of Evidence

After sorting relevant articles that met our keyword search, we removed duplicate news reports on the same suicide incidents in multiple news portals and created a data extraction form using Microsoft Excel (Microsoft Corporation, Redmond, Washington, United States) to further screen data sources and extract data for analysis. KE independently screened and reviewed the full-text newspapers from the keyword search for inclusion. Where there were concerns or conflicts, they were resolved by consensus with the research team.

Data Extraction, Charting, and Data Items

We extracted data from included review articles using a data extraction spreadsheet on Microsoft Excel. Data were extracted and charted for the following title fields: media portal, year, title, state (geopolitical zone/region), method of suicide, sex, age, ethnicity, setting, back story, and primary outcome (suicide completed or not). Data were also extracted using dichotomous items (yes/no) for 12 of the WHO recommendations [1], which included whether the report: (i) educated public about suicide, (ii) sensationalized or normalized suicide, (iii) avoided prominent placement of suicide, (iv) avoided undue repetition of stories, (v) Iavoided explicit description of the method, (vi) avoided labelling site as hot spot, (vii) had a carefully worded headline, (viii) avoided photographs of deceased, (ix) took particular care in reporting suicides, (x) showed due consideration for the bereaved, (xi) provided information on where to seek help, (xii) recognized that media professionals themselves may be affected by stories they report about suicide. 

Data Analysis

We codified and exported all extracted data into IBM SPSS Statistics for Windows, version 28 (IBM Corp., Armonk, New York, United States) for data analysis. We summarized descriptive characteristics of the included reports using frequencies and percentages.

Results

Selection of Data Sources

**Figure 1:**
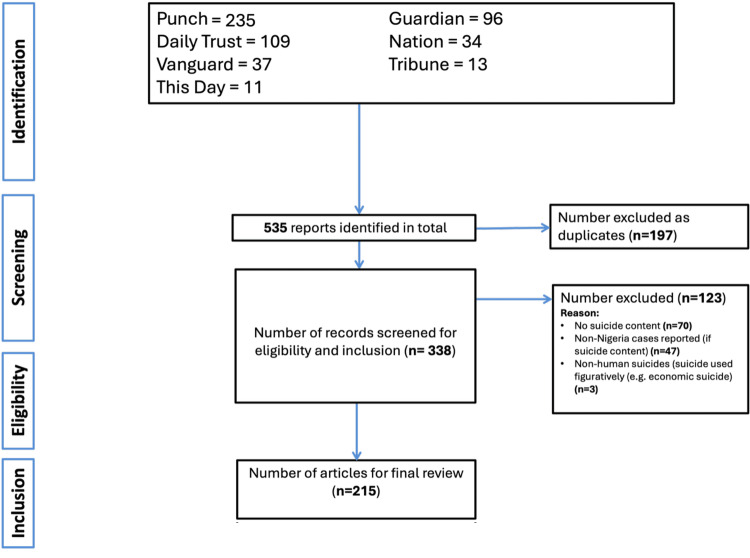
PRISMA flow diagram PRISMA: Preferred Reporting Items for Systematic reviews and Meta-Analyses NOTE: 342 suicide incidents were extracted across the 215 included news reports

Figure 1 shows the PRISMA flow diagram illustrating the process of identifying and selecting news reports for inclusion in this review. A total of 535 reports were retrieved from seven Nigerian newspapers, with the largest share from *Punch* (235 reports), followed by *Daily Trust* (109), *Guardian* (96), *The Nation* (34), *Vanguard* (37), *This Day* (11), and* Tribune* (13). After removing 197 duplicates, 338 unique reports were screened for eligibility. Of these, 123 were excluded for reasons including absence of suicide content (n=70), reporting of non-Nigerian suicide cases (n=47), and non-human or figurative uses of “suicide” (n=3). Ultimately, 215 articles met the inclusion criteria, from which 342 individual suicide events were extracted for analysis.

Data Sources

**Figure 2:**
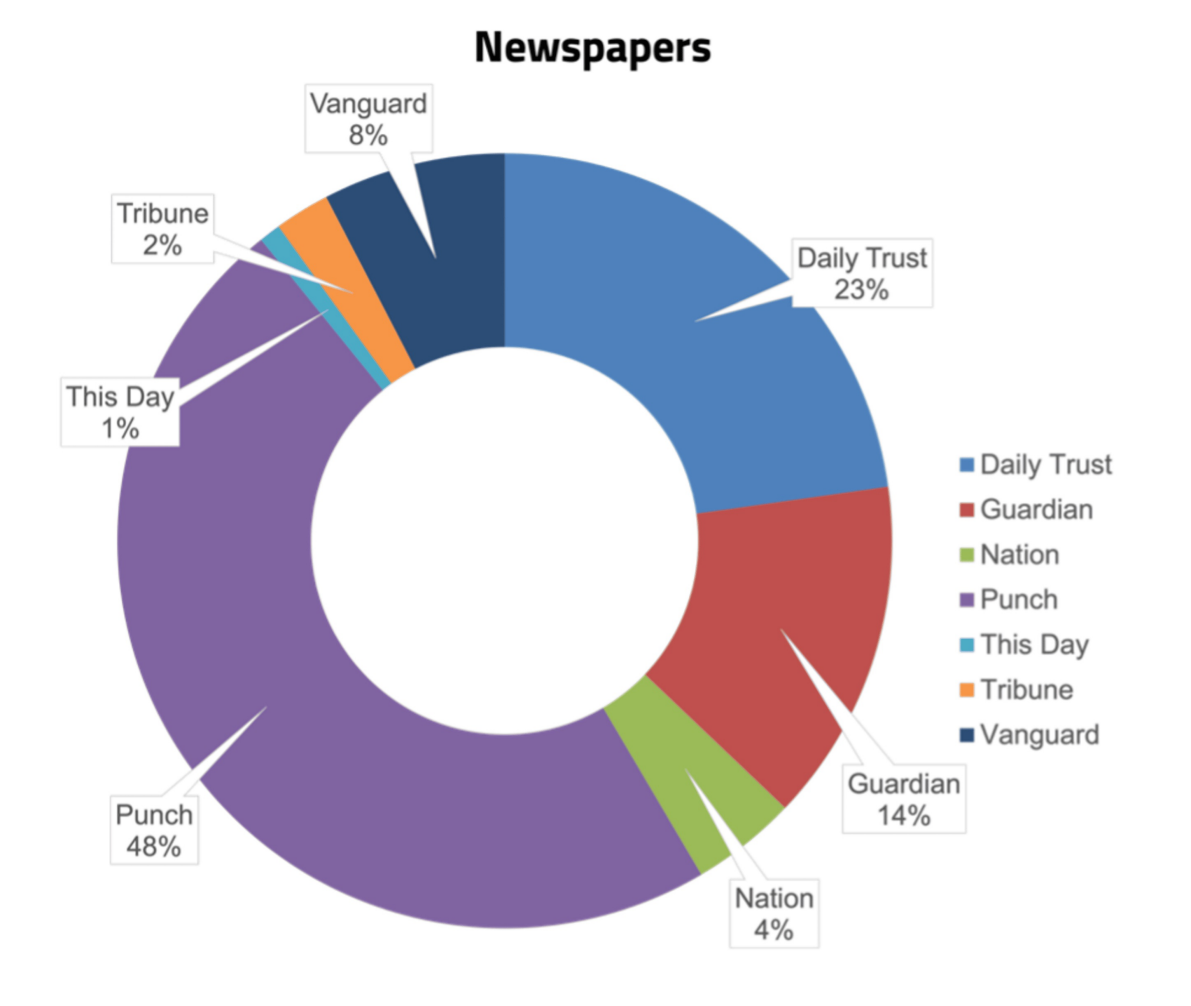
Percentage of reports from the included newspapers

Figure 2 presents the proportion of suicide reports extracted from the seven Nigerian print media outlets included in this study. Nearly half of all reports came from *Punch* (n=103; 48.0%), followed by *Daily Trust* (n=50; 23.3%), and *Guardian* (n=30; 14.0%). The remaining contributions were from *Vanguard* (n=17; 7.9%), *The Nation* (n=9; 4.2%), *Tribune* (n=4; 1.9%), and *This Day* (2; 0.9%). 

Demographic Characteristics 

Between January 2010 and December 2021, a total of 342 distinct suicide events were reported across the selected newspapers, involving 425 individuals. The discrepancy between the number of events (342) and individuals (425) reflects events involving multiple individuals, for instance, suicide pacts or mass incidents (e.g., family poisonings). Among these individuals, 281 (66.3%) were male and 144 (33.6%) were female. Only 17 individuals (3.9%) survived their suicide attempts.

Suicide Method Profiles

Table 1 shows the methods of completed suicide attempts extracted from the reports. Across 342 media‐reported suicide events, suicide bombing was the single most reported method (n=145; 42.3%), followed by hanging (n=87; 25.4%), and poisoning (n=55; 16.0%). All other methods were comparatively uncommon, e.g., gunshot (n=14; 4.1%), drowning (n=13; 3.8%), self-stabbing (n=9; 2.6%), with several appearing only sporadically (each ≤5 events). The "unspecified" category accounted for three (0.9%) reports. Overall, the distribution is heavily right-skewed toward a conflict-related method (suicide bombing), with hanging remaining the leading non-conflict method.

**Table 1:** Suicide methods collected from news reports (N=342 events)

Suicide Methods	Frequency	Percentage
Drowning	13	3.79
Drug overdose	2	0.58
Gunshot	14	4.08
Hanging	87	25.36
Ingestion of corrosive	1	0.29
Jump from height	5	1.46
Jump from moving vehicle	2	0.58
Knife laceration	1	0.29
Poisoning	55	16.03
Set self-ablaze	4	0.17
Slit throat	2	0.58
Self-stabbing	9	2.62
Suicide bombing	145	42.27
Unspecified	3	0.87

Temporal Patterns of Suicide Events

Table 2 reports the temporal patterns of suicide events. Suicide reporting from news reports peaked in 2012 (n=58; 16.9%), with elevated counts through 2014: 47 (13.7%), 2015: 46 (13.4%), 2017: 44 (12.8%), and 2018: 42 (12.2%). Early-decade lows (2011: 3 (0.9%)) were followed by a sharp rise, then a gradual taper by 2020: 26 (7.6%). This pattern suggests period effects in reportage (consistent with surges in events that drew national attention) rather than a steady year-on-year trend.

**Table 2:** Years of occurrence of suicide events (2010 -2021)

Year of occurrence	Frequency	Percentage
2010	14	4.08
2011	3	0.87
2012	58	16.91
2013	19	5.54
2014	47	13.70
2015	46	13.41
2016	15	4.37
2017	44	12.83
2018	42	12.24
2019	29	8.45
2020	26	7.58

Geographic Distribution of Suicide Events

**Figure 3:**
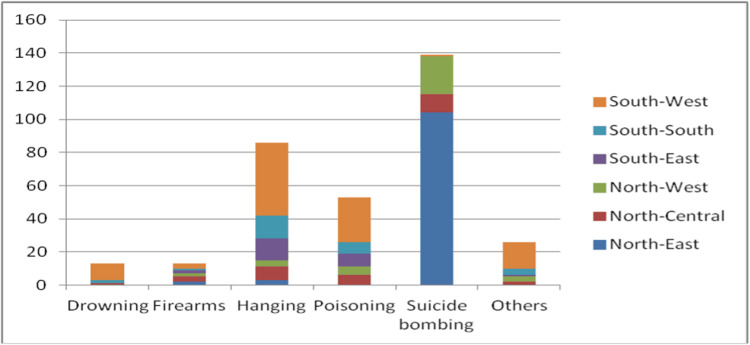
Geographical distribution of suicidal methods by geopolitical zones

Table 3 shows the geographic distribution of suicide events from reports. The geopolitical regions of Nigeria are shown in the Appendices. Events were concentrated in two zones: the North-East, 109 (33.0%), and South-West, 101 (30.6%), which together accounted for nearly two-thirds of all reports. The remaining zones each contributed under 12%: North-West, 37 (11.2%); North-Central, 31 (9.4%); South-South, 28 (8.5%); and South-East, 24 (7.3%). The North-East concentration aligns with the high share of suicide bombing reports (Figure 3), while the South-West concentration likely reflects population/media density and reporting reach.

**Table 3:** Distribution of suicide events by geopolitical zones

Geopolitical zones	Frequency	Percentage
North-East	109	33.04
North-Central	31	9.39
North-West	37	11.21
South-East	24	7.27
South-South	28	8.48
South-West	101	30.61

Gender Differences in Media-Reported Suicide Events

Table 4 summarizes gender-specific patterns in reported suicide events. The mean age of individuals involved was 29.3 years, with women averaging 22.9 years and men 35.6 years, indicating a younger age profile among female individuals who died by suicide. Notably, 47% of suicide events among male individuals occurred in the age group of 11-20 years, suggesting elevated vulnerability in adolescent and young adult males.

**Table 4:** Gender differences with all methods of suicide in Nigeria

Methods	Total suicide event count (n=425), frequency (percentage)	Female (suicide event count =144), frequency (percentage)	Male (suicide event count =281), frequency (percentage)
Drowning	13 (3.06)	4 (2.78)	9 (3.20)
Drug overdose	2 (0.47)	1 (0.69)	1 (0.36)
Gunshot	14 (3.29)	0 (0)	14 (4.98)
Hanging	87 (20.47)	14 (9.72)	73 (25.98)
Ingestion of Corrosive	1 (0.23)	0 (0)	1 (0.36)
Jump from height	6 (1.41)	1 (0.69)	5 (1.78)
Jump from moving vehicle	3 (0.71)	0 (0)	3 (1.07)
Knife laceration	1 (0.23)	0 (0)	1 (0.36)
Poisoning	55 (12.94)	26 (18.06)	29 (10.32)
Set self-ablaze	4 (0.94)	0 (0)	4 (1.42)
Slit throat	2 (0.47)	0 (0)	2 (0.71)
Self-stabbing	9 (2.12)	2 (1.39)	7 (2.49)
Suicide bombing	216 (50.82)	96 (66.67)	120 (42.70)
Unspecified	12 (2.82)	--	--

Among female cases (n=144), suicide bombing (n=96; 66.7%) was most frequent, followed by poisoning (n=26; 18.1%), hanging (n=14; 9.7%), drowning (n=4; 2.8%), and self-stabbing (n=2; 1.4%). Less frequent methods included drug overdose and jumping from a height, each comprising 0.7% of female cases. Among male cases (n=281), the methods included primarily suicide bombing (n=120; 42.7%), followed by hanging (n=73; 26.0%), poisoning (n=29; 10.3%), gunshot (n=14; 5.0%), drowning (n=9; 3.2%), and self-stabbing (n=7; 2.5%).

When comparing male-to-female ratios for specific suicide methods, hanging had a ratio of 5.2:1, indicating a strong male predominance. Jumping from a height had a 5:1 male-to-female ratio. Self-stabbing was 3.5:1 in favor of males. Drowning showed a 2.3:1 ratio. Poisoning was nearly balanced with a 1.1:1 ratio. Drug overdose was evenly reported between genders (1:1). Certain methods, such as gunshot, jumping from a moving vehicle, and self-immolation, were reported exclusively among males. Overall, suicide bombing emerged as the most prevalent method across both genders, with a male-to-female ratio of 1.3:1. 

Media Adherence to WHO Guidelines in Suicide Reporting

**Figure 4:**
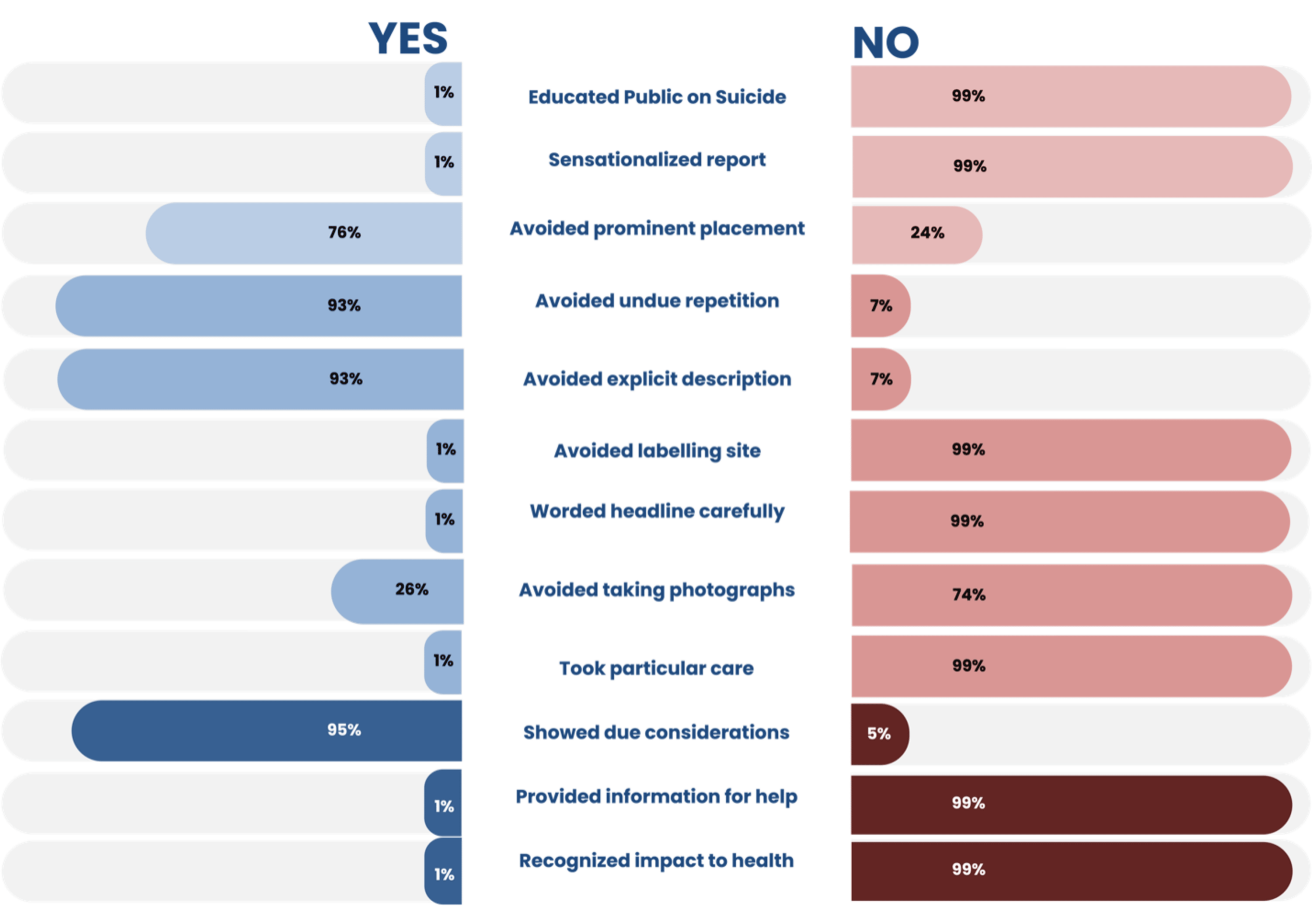
Media reportage adherence to WHO guidelines

Figure 4 presents a detailed analysis of how Nigerian print media adhered to the WHO guidelines for responsible suicide reporting. The results indicate a widespread pattern of non-compliance, with several indicators showing concerning levels of sensationalism and lack of preventive framing.

Among the 342 analyzed suicide reports, 341 (99.8%) articles failed to provide any suicide education or information on where to seek help, such as helplines or mental health services, 341 (99.8%) displayed sensationalist tendencies, often using dramatic or stigmatizing language in headlines and narratives, 259 (75.8%) repeated the word “suicide” prominently and excessively, often in headlines and subheadings, contrary to best practices which recommend minimizing repetition to reduce contagion risk, and 339 (99.2%) explicitly described the suicide method and location, providing detailed and often graphic accounts of the incident. This level of specificity increases the risk of imitation, especially among vulnerable readers. A total of 325 (95.0%) articles did not demonstrate consideration for the bereaved, showing little evidence of empathy or care in framing the impact on surviving families and communities, and 91 (26.7%) included photographs of the deceased or suicide scenes, further compounding the sensational nature of the coverage. Only a negligible proportion of reports adhered to guidelines such as using careful wording in headlines, avoiding detailed visuals, and considering the mental health of media professionals reporting on traumatic events.

Discussion

Our study examined the portrayal of suicide in Nigerian print media from 2010 to 2021, focusing on methods, demographic patterns, regional trends, and adherence to WHO guidelines for responsible suicide reporting. Our findings not only highlight critical disparities in suicide behavior across gender and regions but also reveal substantial gaps in media practice that may hinder suicide prevention efforts in the country.

We included suicide events classified as “suicide bombings” in this analysis due to their frequent identification as such in Nigerian media reports. While we acknowledge the ideological and political dimensions of these acts, we categorized them separately from conventional suicide methods to reflect the media’s framing and to preserve analytical transparency. The predominance of suicide bombing in the North-East geopolitical zone is consistent with the protracted insurgency led by Boko Haram and affiliated extremist groups during the study period [25-27]. Suicide attacks, often involving young males and females coerced into martyrdom operations, have been heavily covered in both local and international media [26]. This concentration of suicide bombings (particularly among women) underscores the weaponization of mental distress and sociopolitical manipulation, where suicide becomes both a personal and political act. These findings align with broader conflict-sociology and terrorism literature, which identifies violent extremism as a driver of both coerced and ideologically motivated self-inflicted death [26,28]. Furthermore, the psychological dynamics underpinning suicide bombing may include identity diffusion and group-induced moral disengagement. These mechanisms, commonly seen among radicalized youth, allow individuals to detach from personal moral codes in favor of collective extremist ideologies. As noted by Pereira and Siqueira, such psychological vulnerability is exacerbated by structural violence and state fragility, conditions prevalent in Nigeria’s North-East [29]. While the WHO suicide reporting guidelines do not explicitly address suicide bombings as a distinct category, suicide bombings are widely recognized in public health and security literature as a form of intentional self-inflicted death, particularly when the individual consents to and executes the act with the knowledge it will result in their own death. We acknowledge that not all suicide bombings may reflect personal suicidality, as some cases may involve coercion, manipulation, or broader ideological motivations. However, we retained these events in our analysis to reflect how the Nigerian media portrays such acts within the discourse of suicide, which has implications for public perception, mental health framing, and ethical reporting practices.

Gender differences in suicide methods were stark as well. Males not only represented two-thirds of all suicide cases but also overwhelmingly used more lethal means, such as firearms, hanging, and self-immolation. Conversely, female individuals, though younger on average, were more likely to use methods such as poisoning and participated disproportionately in suicide bombings, likely reflecting both sociocultural vulnerability and tactical exploitation by insurgent networks [21,30-32]. These patterns reinforce findings from sub-Saharan and global suicide literature, which consistently link method lethality, gender norms, and age-based stressors [8,20,33].

Perhaps most concerning, however, was the media's persistent non-adherence to WHO suicide reporting guidelines. Our findings demonstrated a pattern of sensationalism, lack of preventive framing, and minimal consideration for bereaved families or help-seeking pathways. The fact that nearly all reports failed to provide suicide prevention resources (and often included explicit details of the method) raises ethical concerns. This mirrors the “Werther effect,” whereby irresponsible media coverage may trigger suicide contagion, particularly among vulnerable readers [22,23]. Despite the WHO's 2017 media resource manual [1] and the growing availability of digital media ethics training, Nigerian print media appears to still lag in suicide-sensitive reporting (as at the period of our study). The advent of digital journalism and the 24-hour news cycle, while not captured in our review, likely exacerbates this trend, as online platforms like Instagram (Meta Platforms, Inc., Menlo Park, California, United States), TikTok (ByteDance Ltd., Haidian, Beijing, China), Facebook ((Meta Platforms, Inc.), and X (formerly Twitter; X Corp., Bastrop, Texas, United States) tend to prioritize engagement over ethical framing [34].

However, in recent years (post‑2021), Nigeria has witnessed a growing wave of suicide awareness and mental health advocacy on social media. Youth‑led groups such as the Mentally Aware Nigeria Initiative (MANI) have leveraged platforms like X and Instagram to normalize conversations, share personal recovery journeys, and promote mental well‑being resources [35]. Academic research indicates that digital platforms like X are enabling Nigerian users (especially youth) to challenge cultural taboos and engage in mental health discourse, though persistent stigma remains a barrier [36]. Moreover, newer studies have highlighted that social media influencers play an increasingly important role in shaping mental health awareness among students, although ensuring credibility through partnerships with professionals is essential [37]. While these grassroots efforts may lack standard evaluation methodologies, they collectively represent a cultural shift toward openness in mental health, a trend that presents promising opportunities for aligning with and reinforcing responsible media practices in future public health efforts [14,38-40].

Meanwhile, attempts at suicide remain criminalized under Nigerian law, a structural barrier that not only discourages individuals from seeking help but also reinforces stigma and distorts media reporting [1,10]. Attempted suicide remains a criminal offense in Nigeria under Section 327 of the Criminal Code, punishable by up to one year’s imprisonment. Although prosecutions are rare, documented cases exist, such as *Adeyanju v. State *(one year sentence for overdose) and *Gbadamosi v. State* (six months for insecticide ingestion) [10,41]. Currently, the law does not include any provision for exemption based on mental illness [10]. However, the recently enacted 2023 National Mental Health Act (NMHA) marks a progressive step by recognizing attempted suicide as a psychiatric emergency warranting involuntary hospitalization rather than punishment; however, it stops short of decriminalization [42]. These legal structures continue to serve as a structural barrier, reinforcing stigma, deterring help-seeking, and potentially influencing how the media frames suicide incidents in Nigeria. The NMHA lays the foundation for a rights-based, person-centered approach to mental health care and calls for improved access, protection against discrimination, and the integration of mental health into primary care. While the Act does not yet explicitly decriminalize suicide attempts, it creates a legal and policy framework that could enable future reforms, including decriminalization and ethical media engagement. There is also a growing continental and global movement toward the decriminalization of suicide, anchored in public health and human rights arguments, a direction Nigeria is now better positioned to pursue as part of a comprehensive suicide prevention strategy [34,43,44].

Practical Implications

The findings from our study highlight several actionable steps for improving suicide prevention through ethical and effective media engagement in Nigeria. First, media training is imperative. Regulatory bodies such as the Nigerian Press Council, journalism schools, and professional media associations should integrate WHO or locally adapted suicide reporting guidelines into both foundational curricula and ongoing professional development programs. Embedding these standards will ensure that journalists are not only informed but also equipped to report responsibly on suicide-related stories. Second, collaboration between media houses and mental health professionals should be institutionalized and encouraged. Editorial boards should establish protocols that involve consulting with psychiatrists, psychologists, and suicide prevention experts before publishing sensitive stories. This interdisciplinary approach would enhance both the accuracy and empathy of reporting.

Third, there is a pressing need for continued advocacy toward the decriminalization of suicide attempts. Legal reform would reduce the fear and shame associated with help-seeking, improve the accuracy of suicide reporting, and align Nigeria’s mental health landscape with emerging global standards. The recently enacted National Mental Health Act (2023) offers a promising policy framework within which such reforms can be anchored. Fourth, strengthening data systems and community engagement is crucial. Nigeria’s suicide surveillance is currently fragmented and inconsistent. Partnerships among media organizations, public health institutions, and community leaders could foster grassroots awareness initiatives while also improving the reporting, tracking, and contextual understanding of suicide-related incidents. Finally, digital and social media platforms must be included in future monitoring and capacity-building efforts.

As platforms like TikTok, Instagram, X, and Facebook increasingly shape public narratives (particularly among our youth), it is essential to ensure that ethical messaging and suicide prevention resources are present and accessible in these digital spaces. While Nigeria may not have direct regulatory control over global digital platforms, the government and its regulatory agencies (such as the National Information Technology Development Agency (NITDA) and the National Broadcasting Commission (NBC)) can adopt strategic partnerships and policy frameworks to influence the digital landscape. For instance, the government can: (a) develop national suicide prevention content guidelines and formally engage with social media companies to flag harmful content and promote verified helpline services or mental health campaign, (b) mandate local content moderation offices and algorithmic transparency for platforms operating within Nigeria’s jurisdiction, similar to the European Union’s Digital Services Act model [45], (c) incentivize public-private partnerships to promote mental health awareness through sponsored campaigns, creator collaborations, and community-led digital outreach, especially during high-risk periods (e.g., exam season, economic downturns, or following celebrity suicides), (d) support digital literacy education through the Ministries of Education and Youth, integrating modules on online safety, media resilience, and mental health into school curricula to empower youth with critical thinking and emotional regulation skills.

In addition, Nollywood (Nigeria’s influential film industry) has a unique role to play. Given its cultural reach and storytelling power, Nollywood can serve as a vehicle for destigmatizing mental illness and promoting responsible depictions of suicide. Partnerships between filmmakers, mental health experts, and advocacy groups can ensure that cinematic content is not only compelling but also socially responsible, catalyzing national conversations about suicide and mental wellness.

Study Limitations and Future Directions

Our study presents several limitations that should inform the interpretation of its findings. First, our reliance on print media reports means that the dataset likely underrepresents individuals with a history of multiple suicide attempts and may miss unreported cases entirely. Consequently, the study focuses on completed suicides as they are portrayed in news coverage, not on the full spectrum of suicidal behavior in the population. Second, although the study spans a 12-year period (2010-2021), its cross-sectional design limits the ability to assess how media practices and public responses have changed over time. Third, the reliance on secondary data from newspaper portals introduces the risk of reporting bias, including the overrepresentation of sensational stories and underreporting of suicide attempts or less "newsworthy" cases. Additionally, our study’s temporal cutoff at 2021 may not reflect more recent developments in Nigeria’s media and mental health environment. We acknowledge that in the years since 2021, there has been growing public discourse around suicide decriminalization, the passage of the National Mental Health Act (2023), and increasing visibility of mental health advocacy on social media platforms. These salient shifts suggest that suicide reporting practices (and the broader societal context in which they occur) may have evolved significantly since our study period. As such, our findings warrant cautious, contextually grounded, and temporally aware interpretation, acknowledging the dynamic shifts in media ecosystems, public discourse, and mental health policy in the post-2021 Nigerian landscape. Also, the inclusion of suicide bombings presents definitional complexity, as these acts may reflect ideological motives, coercion, or terrorism rather than personal psychological distress. We included them due to their prevalence in media reporting under the label of “suicide,” but treated them analytically as a distinct category to avoid conflating them with other suicide methods. Finally, while cultural interpretations and socio-religious beliefs strongly influence how suicide is perceived and reported in Nigeria, we did not directly analyse these contextual framings. Future research may address these gaps by incorporating longitudinal digital media analysis, assessing cultural narratives, and capturing post-2021 trends to provide a more current and holistic understanding.

## Conclusions

Our study reveals widespread non-adherence to WHO suicide reporting guidelines in Nigerian print media, characterized by sensationalized headlines, a lack of preventive framing, and the near-total absence of educational or referral content. These reporting patterns not only perpetuate stigma but may also undermine national suicide prevention efforts. To shift this trajectory, it is imperative that both the media and our public health stakeholders recognize the power of narratives. Suicide reporting, when ethically framed, holds the potential to foster awareness, compassion, and prevention. Our study calls for more investment in media capacity-building, suicide decriminalization, improved national suicide data systems, and alignment with global standards for suicide prevention.

## Figures and Tables

**Figure 5:**
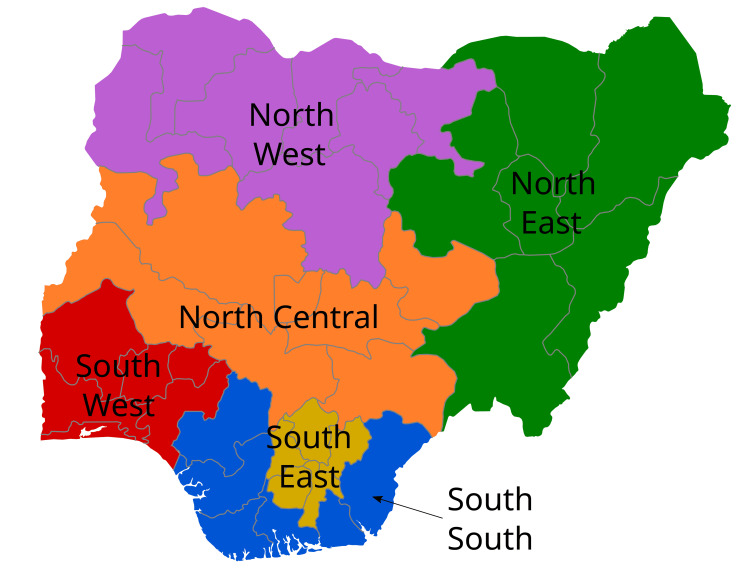
Nigerian map showing the geopolitical zones Image Source: Geopolitical zones of Nigeria [46]; image published under CC0 1.0 license, Universal CC0 1.0 Deed
